# Potentials of Digitally Guided Excursions at Universities Illustrated Using the Example of an Urban Geography Excursion in Stuttgart

**DOI:** 10.1007/s42489-022-00097-4

**Published:** 2022-02-22

**Authors:** Lara Koegst

**Affiliations:** Department of Geography, AG Urban and Regional Development, Eberhard Karls University Tübingen, Tübingen, Germany

**Keywords:** Excursion didactics, Extended reality, Innovative excursion formats, Digital university teaching, Location-based games, Gamification

## Abstract

The main focus of this article is on digital excursion formats at universities and their advantages with and without pandemic-related restrictions on teaching. The development of the application, its didactics and feedback from students are demonstrated using the example of the urban geography excursion developed as part of the 'InExkurs' project at the University of Tübingen. Compared to traditional overview excursions, digitally guided formats allow students more flexibility, autonomy and independence in the implementation. Aspects of gamification and location-based games can be used to increase motivation and, in combination with individual navigation, create associations with scavenger hunts. The use of extended reality on mobile devices enables a diverse application of digital information on site: the use of historical maps and photos as well as the integration of (fictional) characters is exemplified using the developed digital urban geography excursion. The first feedback on the excursion showed that the majority of participating students appreciated the digital format and would like to participate in excursions in this or similar formats more often.

## Abstract (de)

Der Fokus dieses Artikels liegt auf den Vorteilen, die digitale Exkursionen auch ohne pandemiebedingte Einschränkungen der Lehre an Hochschulen haben. Unter der Verwendung von Augmented Reality werden am Beispiel der im Rahmen des Projektes ‚InExkurs‘ entwickelten Stadtgeographie-Exkursion an der Universität Tübingen die Anwendungen und Rückmeldungen der Studierenden aufgezeigt. Im Vergleich zu traditionellen Überblicksexkursionen erlauben digital geführte Exkursionen Studierenden Flexibilität, Eigenständigkeit und Unabhängigkeit in der Durchführung. Aspekte der Gamification und Location-Based Games können zur Motivationssteigerung eingesetzt werden und lassen bei den Studierenden in Verbindung mit individueller Navigation die Assoziation zu Schnitzeljagden entstehen. Der Einsatz von Augmented Reality auf mobilen Endgeräten ermöglicht eine vielfältige Anwendung digitaler Informationen vor Ort: Die Nutzung historischer Karten und Fotos sowie die Integration (fiktiver) Charaktere wird beispielhaft an der erstellten digitalen Stadtgeographie-Exkursion veranschaulicht. Die ersten Rückmeldungen der Teilnehmenden der Exkursion verdeutlicht darüber hinaus, dass das digitale Format von einem überwiegenden Teil der Studierenden Anerkennung findet und diese gerne häufiger Exkursionen in diesem oder ähnlichen Formaten absolvieren werden würden.

## Introduction

In recent years, the development of digital and mobile teaching content in schools and at universities has increased significantly (cf. Witt and Gloerfeld 2018, p. 1) and has received a further boost by the impacts of the Corona virus pandemic. In addition to supplementary digital learning materials, as well as digital teaching and lecture videos, digital and virtual field trips have attracted particular interest in geography (for school students a.o. Pietsch et al. 2020; Stintzing et al. 2020; in a university context among others Forster and Schmid 2018; Schmitz 2021; Seckelmann 2020; for virtual excursions a.o. Budke and Kanwischer 2006). These digital alternatives have some advantages over classical overview excursions: in addition to the increased flexibility and independence of the students, the technology of extended reality in particular opens up possibilities to convey information and impressions to students on site that would remain hidden otherwise (Joan 2015; Stintzing et al. 2020). The potentials of digitally guided excursions and learning content were already recognized and scientifically investigated before the restrictions and conversions to online teaching due to the Corona virus pandemic began to influence teaching. However, trials of digital excursions have so far been limited mainly to school contexts and digital city tours for children (a.o. Hiller et al. 2019). The application of digital excursions at universities has so far only been a selective experiment due to the mostly complex and time-consuming preparatory work by lecturers (Seckelmann 2020, p. 150), nevertheless being developed by e.g. Forster and Hoffmann (2016); Pánek et al. (2018); Schmitz (2021) and Seckelmann (2020) as well as the example used in this paper. In the project 'InExkurs—Innovative excursion formats in a *blended learning format*',^1^ the Urban and Regional Development work group at the Eberhard Karls University of Tübingen developed a digitally guided excursion in Stuttgart in 2020—replacing for the first time the classic urban geography excursion within the Bachelor of Science and Bachelor of Education. Using smartphones as excursion guides students can access content about, as well as locations in Stuttgart implemented in the app 'Actionbound'.

The aim of this article is to take a closer look at the potentials of digitally guided excursions in a university context using the example of the project 'InExkurs' at the University of Tübingen. In addition to the exemplary presentation of the use of the presented digitally guided excursion, a reflection of the excursion and its contents takes place based on the first feedback from participating students. All of this illustrates why digital excursions have great potential for both teachers and students even without the forced use of digital teaching formats due to COVID 19. In this context, the focus of the article is not on the technical implementation of the digital excursion, but rather on illustrating an example of the didactic implementation of a digital excursion guide in a university context that draws on concepts of digital and playful knowledge transfer using forms of extended reality. In the following chapters, after an introductory overview of the advantages of digital excursions and the use of extended reality for teaching, the project ‘InExkurs’ will be presented in order to illustrate the possible implementations of digital excursions using examples from the project and feedback from participating students.

## Why Digitally Guided Excursions Have Great Advantages With and Without COVID

Excursions—"The teaching and learning 'out on site'" (Dickel and Glasze 2009, p. 3)—and the associated work in the field form "the heart of geographical work" (Meyer 2006, p. 134) and are taking on an important role within studies in geography: through primary encounters in the field, excursions enable a vivid transfer of complex knowledge, which is usually taught in abstract form in lecture halls. Moreover, they offer students the chance to understand "the reality already invented by science" (Dickel and Scharvogel 2013, p. 179) which is usually taught in theoretical form in the course of their studies. Therefore, opportunities are created for students to experience spatial developments, and to clarify different constructions of space and allocations of meaning (Dickel and Scharvogel 2013, p. 180), thus contributing to a "demystification” (Lonergan and Andresen 1988, p. 65) of abstract facts. Usually, these excursions take place in the form of classical overview excursions (similar to frontal style teaching), as working excursions or as tracking excursions (germ. Spurensuche), which differ clearly from each other according to the "degree of self-organization of the learning process" (Hemmer and Uphues 2009, p. 39). In all three of these excursion formats, however, limitations of the teaching–learning success can be recognized due to the coincidence of several problems (for more details see Kühne 2019). As already mentioned by Lößner (2011, pp. 27ff.; as well as Rinschede 1997; Schwarz 1995) in the context of school excursions, class size, lack of time, and timetable problems make it particularly difficult to carry out an excursion. These factors can be transferred to excursions at universities: the group size for excursions, especially in the lower semesters, often consists of 25–35 students, which can cause delays, especially in an urban context, as traffic light phases are not adapted to large groups, sidewalks are too narrow, etc., thus complicating the excursion. These circumstances can have a negative influence on motivation as well as on teaching and learning success. Due to practical trainings, block seminars and other activities, as well as diverse subject combinations of future teachers, the planning of excursion dates often collides with other events, often resulting in excursion dates being moved to weekends. Once a date has been found, adverse weather conditions can also make it difficult to carry out the excursion and influence the teaching–learning process (cf. Neeb 2016, p. 242). Furthermore, when selecting a location, the background noise level must be considered (cf. Glasze and Weber 2012, p. 10), which is especially difficult in urban surroundings due to street noise, construction sites, etc. In addition, due to an acoustic overload this is also limiting the "passive-receptive role" (Hemmer and Uphues 2009, p. 43) of students as listeners, especially on overview excursions. Consequently, these difficult conditions during the implementation of excursions result in reduced motivation of teachers and students; it is hardly possible to respond to individual demands and interests, and in overview excursions reproduction predominates rather than the reflection of spatial understanding (a.o. Hemmer and Uphues 2009; Neeb 2016).

With this in mind, digital excursions do not only offer themselves as an advantageous format in times of digital teaching and distance regulations during COVID-related restrictions. Rather, field trips in a digital format offer solutions to the challenges of classical overview excursions in frontal style teaching which were already mentioned: the use of mobile devices contributes to an increased flexibility, as it allows students to plan the implementation individually, independent of time, and thus allowing the adaptation to individual timetables and focal points of interest (among others Arnold et al. 2018, p. 9; Witt and Gloerfeld 2018, p. 2). This applies in particular to the presented example as students have the possibility to choose from thematically different app-guided stations. In addition, the use of headphones as well as the use of visual media can reduce the influence of background noise. Since 99% of all young people already have access to smartphones (Feierabend et al. 2018, p. 6) it can be assumed that the proportion is similarly high in students, therefore making the smartphone a digital end device that is generally available without further effort. A possible distribution of the excursion over two or more days also offers the possibility to meet the needs of the students, to counteract the loss of motivation towards the end of longer excursions, as observed by Neeb (2016, p. 335f.), and to adapt to possible adverse weather conditions. Furthermore, the implementation in small groups with a digital device facilitates the movement in an urban context; in combination with playful tasks, students are additionally encouraged to reflect on what they have learned and to develop their own perspectives, thus no longer participating as mere listeners (cf. among others Hemmer and Uphues 2009). During the development of the field trip, however, it must be kept in mind that the information provided on the smartphone should not lead to a sensory overload of the students or distract from the content being taught. According to Seckelmann (2020, p. 151), especially audio files have proven to be appropriate, as they are less distracting from the surroundings than texts, images or videos. While the use of the smartphone serves as a digital excursion guide, it should complement the physical environment with the right amount of information rather than distracting from it. By integrating the digitally guided excursion into a lecture or seminar, it can be followed up and any questions can be discussed, so that the missing option of asking a lecturer questions during the excursion can be compensated (cf. Seckelmann 2020, pp. 159–162).

The use of mobile digital devices also allows the implementation of elements of game-based knowledge transfer through gamification and location-based games, which can have a motivational and attention-boosting effect on participants in educational contexts having attracted scientific attention in recent years (among others Deterding et al. 2011; Deterding 2014; Liebermann 2009; Michael and Chen 2006; Pánek et al. 2018, p. 286). Gamification refers to the integration of "game design elements in non-game contexts" (Deterding et al. 2011, p. 10). The concept is particularly applied in everyday school life (cf. Pietsch et al. 2020, p. 37), but it is also integrated in higher education, for example on the basis of quantifiable results that promote conscientious completion through extrinsic motivational moments (Pánek et al. 2018, p. 287). Location-based games are particularly suitable for excursions, as they "can be created and used in educational environments, in ways that promote and engender playful forms of place-based knowledge production." (Pánek et al. 2018, p. 287). The physical location of the participants is integrated into the game accordingly (cf. Jacob and Coelho 2011; Pánek et al. 2018, p. 287), so when a location determined by GPS is reached, location-related information is activated on the digital device (Pánek et al. 2018, p. 287). Furthermore, according to Nicklas et al. (2001, p. 61f.) a distinction can be made between "mobile games" (no GPS tracking necessary), "location aware games" (activation occurs when a position determined by GPS is reached) and "spatially aware games" (enables integration of the physical environment of the participants, activation occurs when a spatial context is reached). The excursion considered here is based on the category of location aware games (cf. Nicklas et al. 2001, p. 62) as reaching certain GPS determined stations to activate information is integrated into the excursion.

## Extended Reality in (High School) Teaching

**Fig. 1:**
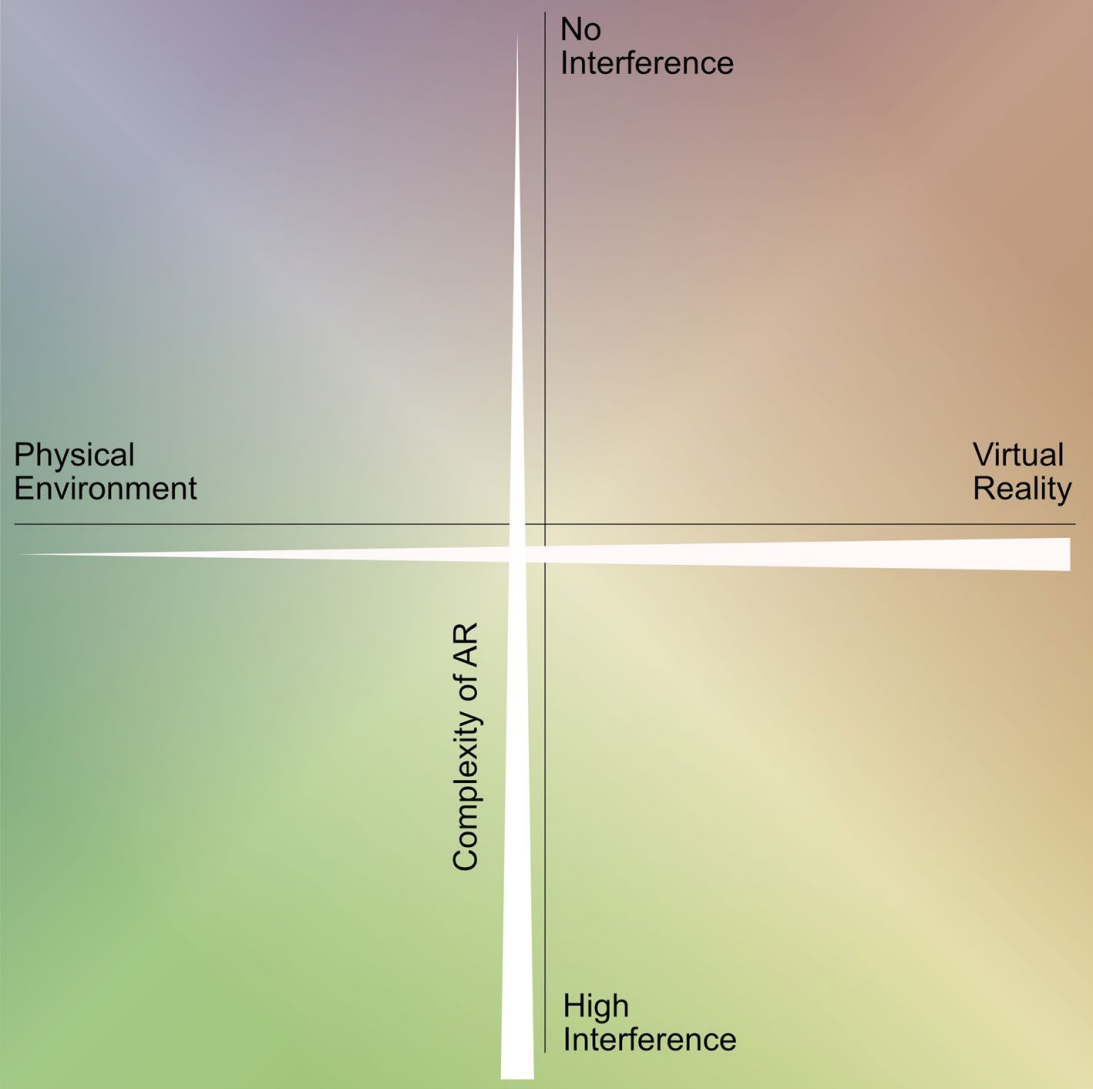
The dual polarity of the complexity of extended reality: hybridizations are found between entirely physical as well as entirely virtual environments, as well as varying degrees of interference between the two. The form of extended reality applied here exhibits only minor interference, but supplements the physical environment with virtual content via the student’s smartphones (Own representation based on Milgram et al. (1994, p. 283) and Kühne et al. (2020, p. 6)

Extended realities and their potentials for visualizing geographic information have been studied increasingly in recent years (among many: Çöltekin et al. 2020; Dickmann et al. 2021; Dörner et al. 2019; Jung et al. 2020; Kersten and Edler 2020; Kühne 2021; Liu et al. 2021) and is opening up more opportunities in teaching through its integration into schools and universities (among many: Arici et al. 2019; Avila-Garzon et al. 2021; Bacca et al. 2014; Challenor and Ma 2019; Dunleavy et al. 2009; Joan 2015; Kljun et al. 2020; Klopfer and Sheldon 2010; Lindner et al. 2021; Prisille and Ellerbrake 2020; Wu et al. 2013). In particular, augmented reality "is uniquely changing the way people learn with mobile devices" (Joan 2015, p. 9) and thus also provide new teaching methods in excursion didactics. As described by Budke and Kanwischer (2006; likewise, Hamilton et al. 2021; Zhao et al. 2020b; Prisille and Ellerbrake 2020; Radianti et al. 2020), the application of virtual reality in geographic teaching also shows great potential. However, augmented reality methods are ideally suited for excursions in which pupils and students are given the opportunity to form individual impressions on site in accordance with constructivist excursion approaches: the physical environment on site is supplemented with virtual information—not replaced by them, as it would be the case with virtual reality (cf. among others Wu et al. 2013, p. 42; Azuma 1997)—so that, among other things, abstract concepts and complex spatial processes can be made comprehensible as well as the conveyance of phenomena that are usually not observable with the naked eye in the physical environment (Wu et al. 2013, p. 41; Arvanitis et al. 2007; Klopfer and Squire 2008). Moreover, students learning performance has shown to be enhanced “when mere passive intake of information is replaced by active experience and learning by doing” (Lindner et al. 2021, p. 255; Clark and Mayer 2008; Reeves 2012). The focus of the excursion considered here, as in the excursion described by Klopfer and Sheldon (2010, p. 86) is on learning in "real-world contexts that are lightly augmented using digital information from mobile devices like cell phones", so that the students' interaction is primarily with their environment on site and is only supplemented by digital information (e.g. historical photos, maps; information texts and tasks that promote interaction with their surroundings). This relationship between the physical and virtual environment can be defined as a dual polarity (cf. Kühne et al. 2020, p. 6): as described by Milgram and Kishino (1994, p. 1321; also, Milgram et al. 1994, p. 283), the physical environment and the virtual environment stand opposite each other, with different degrees of hybridization in between, locating augmented and mixed reality here (see Fig. 1). The complexity of augmented reality can be further differentiated on the basis of interference, the overlaying of the physical environment with virtual elements. No direct interference, as for example in the digital urban geography excursion presented here, is working with, e.g., the provision of virtual data on smartphones (without an overlay using the camera), while at a high level of interference, the virtual objects can be assumed to react to the physical environment. In regard to this complexity of extended realities, the concept applied in the developed digitally guided excursion represents—according to the mentioned broad definition of augmented reality—a light form of augmented reality with little to no direct interference, although still adding varying virtual information to the surrounding physical environment of the participants via the smartphone display.

According to Wu et al. (2013, p. 42) the understanding of augmented reality (AR) in a broad sense has shown to be viable in educational applications, as this definition "suggests that AR could be created and implemented by various technologies, such as desktop computers, handheld devices, head-mounted displays and so on" (Wu et al. 2013, p. 42; Broll et al. 2008). Consequently, any technology that purposefully mixes virtual information with information from the physical environment can be called augmented reality (cf. Wu et al. 2013, p. 42; Klopfer 2008). Initial findings from spatial cognition research also indicate the need to take cognitive mechanisms of action into account that arise from the representation of augmented reality and—as further development of the *cognitive map design research* established since the 2000s (cf. Montello 2002)—to develop design rules that counteract cognitive distortion effects (e.g. distorted distance estimates or inaccurate positioning; Dickmann et al. 2013; Keil et al. 2020; Tversky 1981). As a technical basis for the combination of virtual information and the physical environment, mobile devices equipped with mobile internet and GPS enable individual and flexible learning and can enhance the interaction with the environment in location-based applications (cf. Broll et al. 2008; Zhao et al. 2020b; Wu et al. 2013, pp. 43ff.; Zhao et al. 2020a, p. 1342).

Furthermore, the use of extended reality creates the possibility of visualizing things and processes that are not usually visible (a.o. Wu et al. 2013, p. 44, Arvanitis et al. 2007). These can include historical city views, physical processes, or fictional objects. Objects and processes below the earth's surface can also be visualized using extended reality techniques. (Stylianidis et al. 2020). As an example, it is possible to integrate interaction with virtual characters who appear location-dependently, thus giving students insight into other perspectives and simulating expert discussions (cf. a.o. Stintzing et al. 2020; Pietsch et al. 2020). Likewise, it is possible to include digital digressions in the on-site observations, such as the analyzed discussion by Kühne et al. (2021) on the causes and effects of the storm and floods in western Germany in July 2021 which can be integrated into the consideration of the impacts on physical space on site. Accordingly, augmented reality makes an important contribution to bridging the gap between formal and informal learning contexts and the associated increase in intrinsic motivation (a.o. Wu et al. 2013, p. 43f.), which ultimately results in positive effects on students' learning interest (Zhao et al. 2020a, p. 1342). Furthermore, with the use of augmented reality, participants are provided with a platform through which they can access virtual content (e.g., images, texts, videos and audio files, but also location-based information) that enables them to explore and investigate their physical environment in an information-based way (Wu et al. 2013, p. 43; Dede 2009).

## The Project "InExkurs" as an Example of a Digital Excursion for Geography Students

Based on these advantages of a digital excursion and the potentials of augmented reality for teaching and especially excursions, a digital excursion was developed in 2020 by the Urban and Regional Development Working Group at the University of Tübingen under the direction of Olaf Kühne, which included urban geographic topics in Stuttgart for students in the second semester of the Bachelor of Science and Bachelor of Education in Geography. In the following chapters, the main features of this project are first presented, then its implementation is exemplified and finally supplemented by the first feedback provided by participating students.

### Outline and Framework of the "InExkurs" Project

**Fig. 2:**
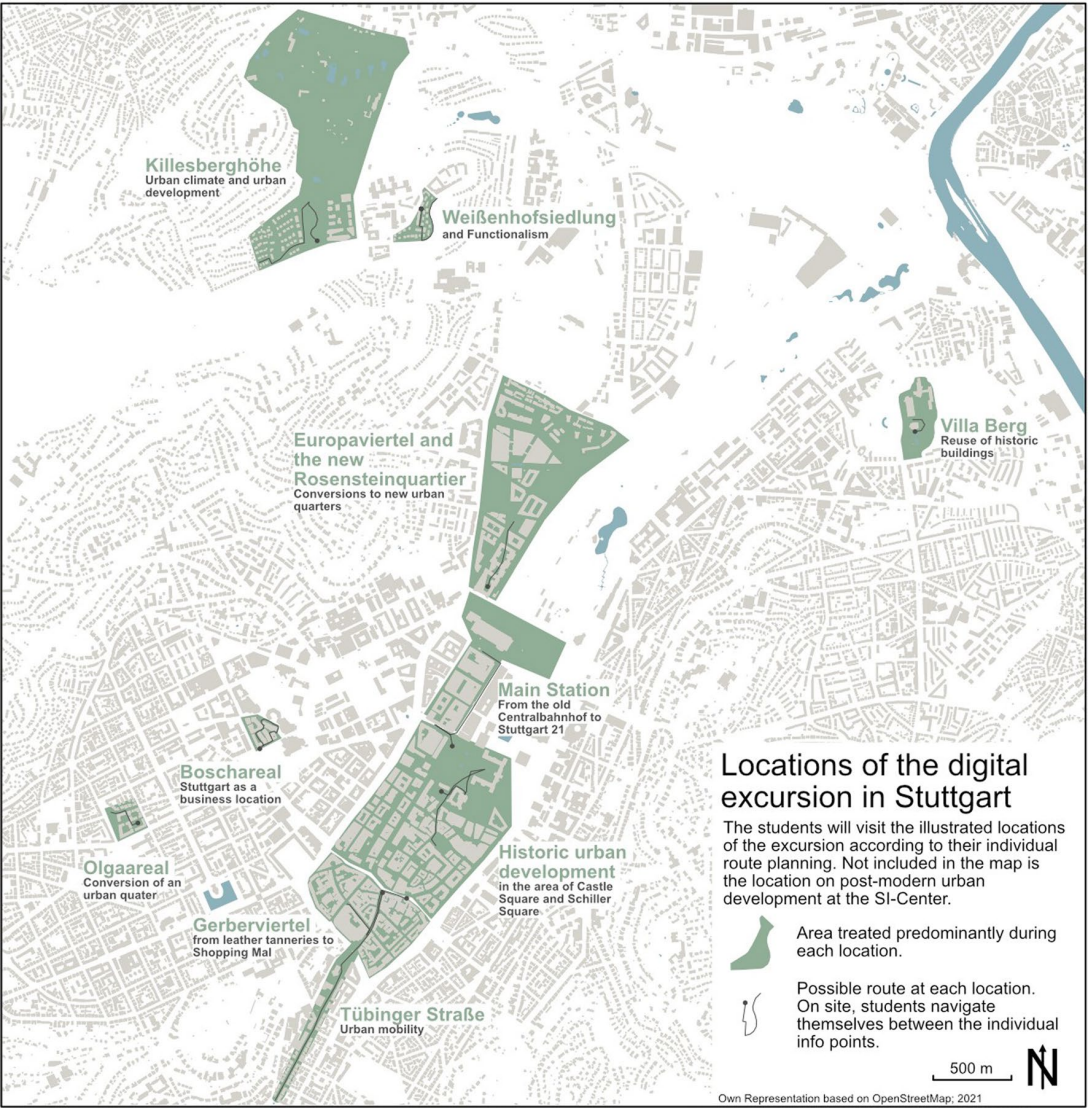
Locations of the digitally guided excursion sites in Stuttgart. Due to its location outside the city center, the location at the SI Centre where the students work on the basics of postmodern urban development is not included in the illustration. Here it became apparent that the distance of the location from the other excursion sites in the inner city meant that the site was only visited by a small proportion of the students. Therefore, when creating an excursion route with flexible site selection by the students, attention should also be paid to the spatial proximity of the individual sites. (Own representation based on OpenStreetMap, 2021)

The development of the urban geography excursion considered here was based on the project 'InExkurs—Innovative Exkursionsformate im *blended-learning* Format', which was funded by the Baden–Württemberg Foundation and the Stifterverband as a senior fellowship in the program "Fellowships for Innovations in Higher Education". Arnold et al. (2018, p. 142f.) defined the implemented concept of blended learning as the aim "to use the advantages and avoid the disadvantages of different elements in the organization of educational offers by mixing them", such as the applied combination of lecture and seminar in classroom teaching (during the restrictions due to Corona, however, taking place in asynchronous and synchronous forms of online teaching) with the digital, smartphone-supported excursion in Stuttgart, which accordingly avoids the problems of classical overview excursions. The contents of the excursion were developed in cooperation with students of the master program 'Human Geography/Global Studies' in the course ‘GEO 82—Geography of Urban Structures and Processes’. In a way this can be seen as a double learning success, as both Bachelor and Master students can gain additional value from developing and participating in the excursion. Technically, the digital excursion is based on the app-based application 'Actionbound', which is often used in the educational sector for digital scavenger hunts and rallies due to its multimedia and flexible application possibilities (Hiller et al. 2019, p. 18; Hermes and Kuckuck 2016). So-called 'bounds' are created to convey content, which can be accessed and used on site via a QR code. In this form of serious-games, participants receive points for each successfully completed task, which can have an increasing effect on motivation. Each site of the excursion is explored by a separate bound and structured via predefined locations that must be reached to unlock further information and tasks. The students have the opportunity to independently put together an excursion route with at least seven stations from a total of eleven thematically different bounds according to their own interests, thus promoting individuality and interest (cf. also Wu et al. 2013, p. 44; Klopfer 2008; for the general meaning of space and landscape in games see also Kühne et al. 2020). The different sites of the excursion which the students can choose from and put together their individual route are shown in Fig. 2.

The development of the excursion followed the design-based research approach developed and implemented for a digital city rally by Hiller et al. (2019; further: Design-Based Research Collective 2003). Based on the urban geography content of the lecture, topics wich should be covered in the excursion were identified and corresponding locations to illustrate the topics practically in relation to the development of Stuttgart were defined in a first step. In addition to the teaching and application of urban geographic contents as an addition to the lecture and seminar, the digital excursion also intends to convey different understandings of space and landscape (for more details see Koegst et al. 2022). Next, in the summer semester of 2020, the master students developed the contents of the individual stations, and prepared and implemented them in Actionbound. Subsequently, the created bounds were examined regarding their applicability, revised if necessary, and formally standardized. The herby created test version of the excursion was tested by students of the bachelor course. Based on the feedback received, the excursion and its technical implementation in Actionbound were further optimized to ensure that the excursion was as good and practicable as possible for the first run. After this first excursion on a larger scale, the participating students had the opportunity to record their experiences and comments in an evaluation form and thus contribute to further optimization. The practicable comments gained this way were finally incorporated into the excursion for a last optimization. The preparation effort is accordingly significantly higher than for classic overview excursions—Seckelmann (2020, p. 150) mentions "per hour excursion approx. 10" hours of preparation effort. However, this justifies itself by less preparation effort in the following excursion runs. Moreover, the development of contents by master students the work load is reduced, however, the tasks of optimization and standardization remain. The implementation of the urban geography content is exemplified in the following paragraphs, followed by a summary overview of the students' feedback after the first implementation of the excursion.

### Examples of the Urban Geography Excursion in Stuttgart

**Fig. 3:**
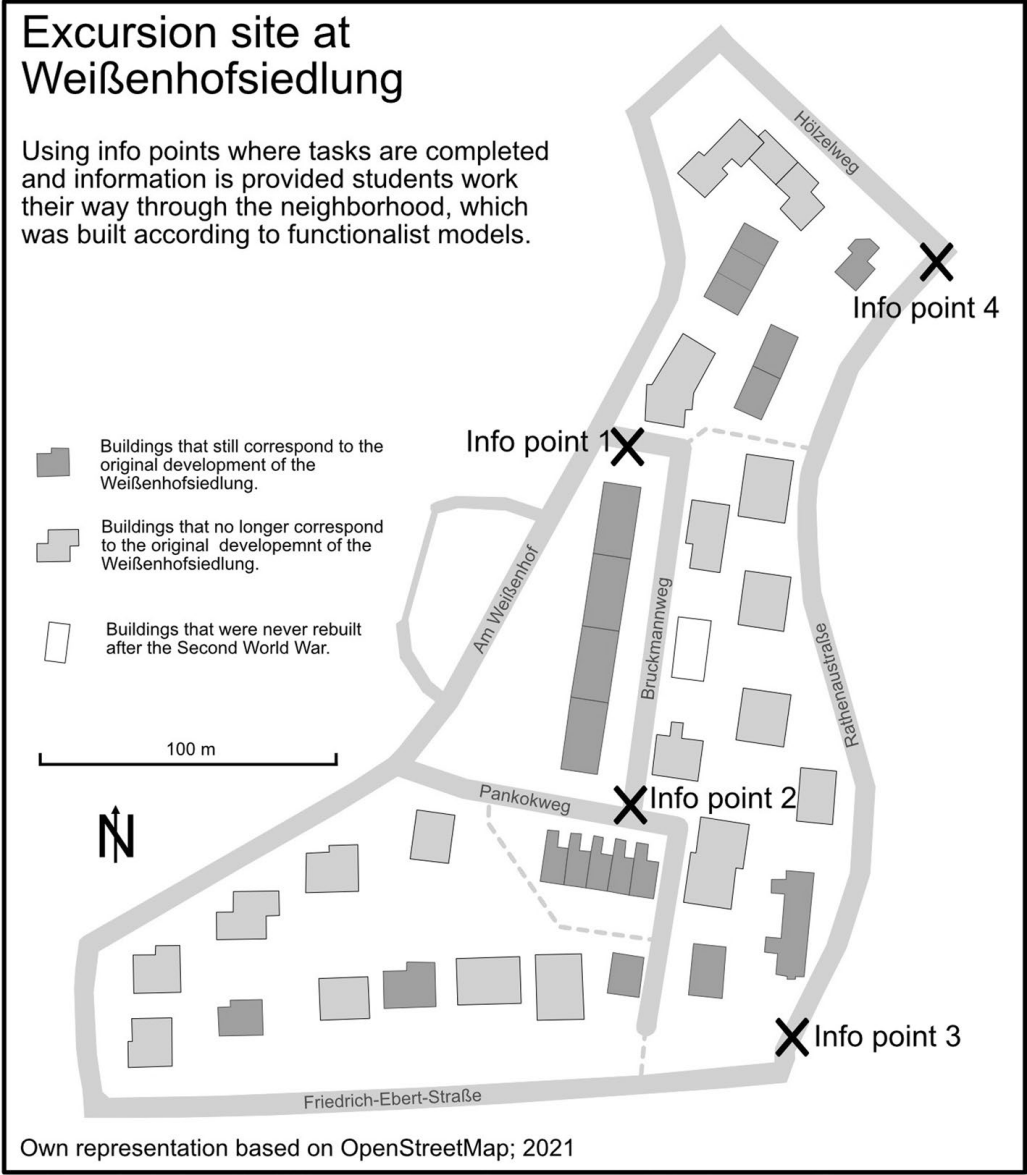
At the individual excursion sites, the students are guided through the area by a route consisting of different information points and are encouraged to engage with the space on site through various tasks. For example, on the route through the Weißenhofsiedlung shown here, students have to identify structural features of functionalism on site integrated in a multiple-choice task. (Own representation based on OpenStreetMap, 2021)

The implementation of the content in Actionbound is possible in a variety of ways (cf. Koegst et al. 2022). An exemplary overview of conveyed topics, target skills and exercises is provided in Table 1. In addition to information texts, various task formats (for example, multiple choice, drag and drop, recording an audio or photo, estimating a number) enquire the students about taught theories and the surrounding environment, which, in addition to applying what has been learned, encourages them to reflect on and question their own perceptions and the theories associated with them. For each successfully answered question, the students receive points, which result in a ranking at the end of the individual stations, ultimately having a motivational effect through the integration of playful gamification elements. Points are also awarded for reaching specified locations, whereupon location-based information is unlocked. With the help of a map provided in the app, students navigate between the individual info points of the excursion sites and must find their way around Stuttgart. Figure 3 shows an example of the students' route through the Weißenhofsiedlung; in the app, the students navigate independently using an OpenStreetMap map integrated in the application.

**Table 1:** Exemplary overview of the conveyed geographic topics, target skills students should learn on site and the respective method, exercise, and supplementary digital materials used. All exercises are done in small groups

Excursion site	Conveyed geographic information	Example of the sites target skills	Methods/supplementary digital material/exercises
Weißenhofsiedlung	Functionalist urban development, Charta of Athens	Comparing different architectural styles and their societal background	Discussing the differences of the functionalist Weißenhofsiedlung and the nearby traditionalist Kochenhofsiedlung based on impressions gained on site and taught contents, recording the group’s results
Castle square	Historic urban development of Stuttgart	Drawing conclusions about Stuttgart's historic urban development based on on-site impressions and taught information	Localization of the current location on a historical map; Comparing the surroundings with a historic photo to identify structural and architectural differences and inferring their origins (see, e.g. Fig. 4)
Olga-Areal	Conversion of urban neighborhoods and their new development	Understanding and empathizing with different political and social perspectives and demands regarding the development of urban neighborhoods	Through the use of different fictional characters, varying political and social expectations are presented, illustrating an expert discussion
Gerberviertel	Historic development and inner-city shopping center	Categorizing and mapping information gained on site	Based on the shopping centers floor plan, mapping the different types of shops and uploading a picture of the drawn map

Own representation based on the developed excursion

**Fig. 4:**
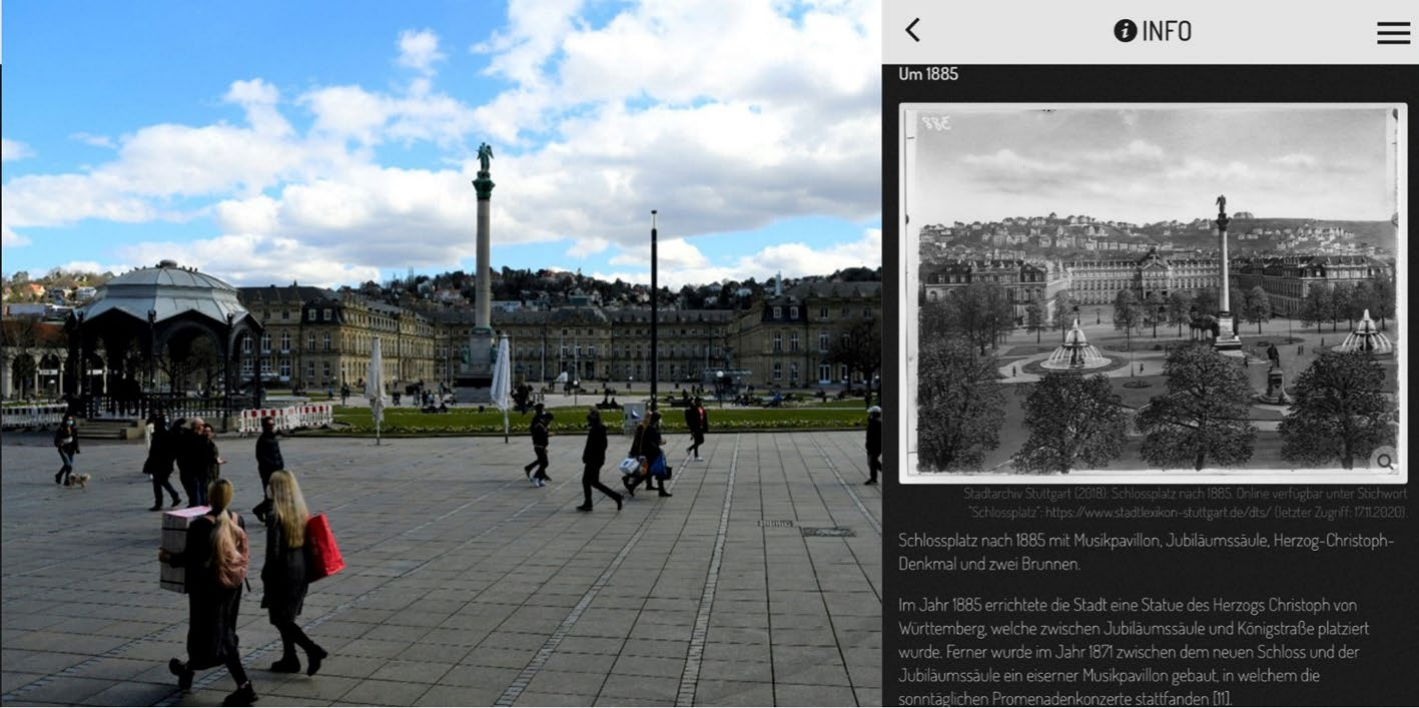
Example of the integration of a historical photograph: students have to relate the information provided to impressions gathered on site and identify the design differences (left: own photograph 2021; right: own representation in Actionbound; image source: Stadtarchiv Stuttgart 2018)

A closer examination of the lecture taught contents and the environment on site can be achieved, for example, by working with (historical) maps. Here, the use of the smartphone as a mobile excursion guide is particularly helpful, because digital maps can be enlarged as desired and facilitate the handling in comparison to paper maps in large groups of the traditional overview excursions. On site, working with the map helps to promote a sense of direction and encourages students to engage with their surroundings. Locating tasks on historical maps are particularly suitable for making students aware of the changes in their environments and visualizing structural or planning changes (cf. Neeb 2016). Small-scale changes in the students' surroundings can also be illustrated particularly well through the direct juxtaposition with historical photos on a smartphone. For example, students are asked to identify the changes in the design of the Schlossplatz (castle square) by comparing the historical photo with their view on site (see Fig. 4 and Table 1). At the excursion site at Boschareal, students are provided with information on the sites past industrial use as well as a historical plan of the former factory complex. Based on this information, the students are asked to search for traces of the former use that are still evident, even after the complex’s conversion (cf. among others Hemmer and Uphues 2009). This is supposed to sensitize the participants to pay attention to easily overlooked traces in their surroundings and to draw conclusions about the development of buildings or a city based on these clues.

Incorporating aerial photography into the digital field trip allows students to take in a perspective that would normally remain hidden. For example, at the Killesberghöhe site, an aerial view is provided of the area of the former Stuttgart Trade Fair Centre, which is now occupied by the Höhenpark Killesberg (a new public park) and two new city districts. Using the aerial photograph, the students can gain an impression of the former use of the site and are encouraged to compare it with the current use, which subsequently contributes to a better understanding of the development of their surroundings (cf. among others Klopfer and Sheldon 2010). The task of locating oneself on the aerial photograph of the former trade fair also requires an intensive examination of their current surroundings and emphasizes the size of the area.

Another playful aspect and, furthermore, a form of gamification enabled by the use of augmented reality is the integration of (fictional) characters. These appear in the course of the excursion in relation to the student’s location and are used to illustrate different perspectives. For example, the students meet Mies van der Rohe, the architect in charge of developing the Weißenhofsiedlung. Written in the form of a letter, students learn about considerations and contexts during the planning and construction of the settlement and receive an explanation of the most important aspects of functionalist urban design. In the Olga-Areal (a converted residential complex), different political perspectives are conveyed regarding the conversion and development of an urban neighborhood in Stuttgart: The perspective of a fictitious city councilor is supplemented by the perspective of a representative of a fictitious citizens' initiative, both illustrating different ideas of the neighborhood’s development. Moreover, this implementation of fictious characters is giving participating students the impression of an expert discussion and promotes understanding and empathizing with varying political and social perspectives.

### First Feedback from Participating Students on the Digitally Guided Excursion: "Because Scavenger Hunts/Escape Rooms Are So Much Fun and You Learn So Much"

Based on an evaluation form filled out by the participants after they completed the digital excursion (*n* = 74), a detailed opinion of the students about the digital implementation as well as experiences during the implementation could be established. Overall, the digital format of the excursion can be considered a success, as 72.4% of the participants stated that they would like to participate in digital excursions in this or similar formats more often. Reasons for this are consistent with findings from other publications (among others Witt and Gloerfeld 2018; Arnold et al. 2018): increased flexibility, autonomy, the opportunity to explore places independently and not being dependent on the pace of a large group and the explanations of an excursion leader. In contrast, 27.6% of the students were rather less convinced by the digital excursion: while 15.5% of the participants accepted the digital excursion only as an alternative during the pandemic-related restrictions, 12.1% of the students stated that they missed the guidance of lecturers who were available for questions. Others interpreted precisely this absence of lecturers positively, as there was no dependence on the group, illustrated by the following statement of a participating student: "I would definitely want to conduct excursions in this format even without COVID 19. With excursions in a large group, it gets boring and tedious more quickly because you always have to keep up with the time of the slowest person. With this excursion, you were able to set your own pace, and you were free to manage your time on site. You could also determine the time of the excursion yourself, which is of course better even without COVID, because you often have other appointments and events during the week and at the weekend and you can therefore plan the excursion better.”

The students are divided among each other about a distraction from the environment due to a focus on smartphones as the carrier of digital information: 40.5% of the participants could not be distracted, whereas 37.8% of the participants stated they were sidetracked. This can be explained by a high proportion of information texts and can be counteracted by an increased use of auditory information. 77% of the participants also assessed the combination of on-site observations with digital information on the smartphone positively. This shows that the transmission of information using augmented reality is welcomed and accepted and represents an important pre-condition for the independent and autonomous implementation of the excursion. In this context, not only the use of visual information turned out to be purposeful, but also linking it with acoustic information transfer seems to be profitable for the students. This feedback from participating students thus confirms the achievement of the aim to bridge formal and informal learning contexts through the use of augmented reality (cf. a.o. Wu et al. 2013, p. 43f.).

Most of the participants had no difficulties with technical handling, but high battery consumption and difficulties with GPS location were reported. The latter poses problems, as the delayed recognition of a reached location and the resulting delay in receiving points causes unwillingness, which counteracts the original motivation-increasing goal of this gamification element (cf. a.o. Wu et al. 2013). Nevertheless, 83.7% of the students stated that they had enjoyed the digital excursion, “because scavenger hunts/escape rooms are so much fun, and you learn so much". This also illustrates the successful application of the gamification elements, which turned an excursion into an educational and informative scavenger hunt and the students' interest in learning—as Zhao et al. (2020a, p. 1342) postulated– is influenced positively.

## Conclusion—"It Makes the Excursion More Independent and Flexible"

Compared to traditional overview excursions, digital excursions have several advantages that enable students to acquire knowledge in a more independent, flexible and autonomous way, creating a direct link between what they have been taught and the impressions they experience on site. The integration of elements of gamification and location-based games contribute to an increased motivation of the students and thus promote the consolidation of lecture contents. The use of extended reality allows for the integration of a variety of information that complements students' on-site perceptions with further interesting and educational digital information. In particular, historical photos, aerial photographs and (historical) maps as well as (fictional) characters contribute to an increased engagement of the participants with their surroundings and promote reflection on what has been learned. The feedback after the first implementation of the urban geography excursion in Stuttgart, which was developed within the project 'InExkurs', makes it clear that the majority of the students approve of the more flexible, independent and autonomous digital format. Especially the possibility of designing the route according to individual interests met with approval among the students. Furthermore, an increased incorporation of audio files will, in a further optimization of the excursion, reduce the focus on the smartphone through the previously necessary reading of information texts, thus allowing students to directly connect visual impressions on site with the acoustic knowledge transfer. Digital excursions, with the mediation of information through extended reality, are able provide information, which would otherwise remain hidden. The format therefore offers great potential not only for geographic teaching at universities but can also be a profitable opportunity in other subjects to interrupt formal learning, convey information and applying it practically.

## Footnotes

1 The project 'InExkurs—Innovative Exkursions Formate im *blended-learning* Format*'* was developed in 2020 under the direction of Olaf Kühne at the Department of Geography of the Eberhard Karls University of Tübingen as part of the program "Fellowships für Innovationen in der Hochschullehre" (Fellowships for Innovations in University Teaching) as a senior fellowship funded by the Baden-Württemberg Foundation and the Stifterverband and was tested for the first time in the summer semester 2021 in the module 'Geo 22—Urban Geography' of the Bachelor of Science.
